# What is the existing evidence base for adult medical Same Day Emergency Care in UK NHS hospitals? A scoping review

**DOI:** 10.1016/j.fhj.2024.100011

**Published:** 2024-02-20

**Authors:** Sue Dean, Julian Barratt

**Affiliations:** a Same Day Emergency Care, Pilgrim Hospital, Boston, United Lincolnshire Hospitals NHS Trust, UK; b School of Health Sciences, Faculty of Medicine and Health Sciences, University of Nottingham, UK; c Faculty for Advancing Practice, NHS England Workforce, Training and Education, Midlands, UK; d Centre for Advancing Practice, NHS England Workforce, Training and Education, England, UK; e Faculty of Education, Health and Wellbeing, University of Wolverhampton, UK; f Aston Medical School, Aston University, Birmingham, UK

**Keywords:** Same day emergency care, SDEC, Ambulatory emergency care, AEC

## Abstract

**Introduction:**

This scoping review focuses on acute medical Same Day Emergency Care (SDEC), as medical patients represent a significant proportion of emergency admissions in the NHS.

**Methods:**

This scoping review has been conducted in accordance with the JBI methodology and as detailed in the published scoping review protocol.

**Results:**

Identified papers included two observational cohort studies, four audits, four review articles, two opinion pieces, a paper on potential future developments, three policy documents, one strategy paper and a position statement. Key themes were extrapolated and discussed in a narrative.

**Conclusions:**

The scarcity of literature in relation to the quality, safety, and effectiveness of SDEC highlights the need for further study. Therefore, there is a pressing need for SDEC specific research within the UK.

## Introduction

Building on previous work undertaken within ambulatory emergency care (AEC), to support non-admitted emergency care pathways, Same Day Emergency Care (SDEC) is a new model of care that has been developed within the NHS,^1^ as mandated in 2019 by the NHS Long Term Plan;^2^ the Same Day Strategy for SDEC was subsequently released in 2021.^3^

The model suggests that SDEC can meet local health needs by providing an alternative to emergency department (ED) attendance or hospital admission. SDEC services are considered essential to the future provision of acute care, in an aging society with limited health care resources.^3^

However, as with AEC, considerable service variation exists within the SDEC model. Some organisations offer a broad range of specialties within their SDEC services: paediatric, frailty, gynaecology, surgery, oncology, in addition to acute medicine, whereas other organisations focus their SDEC model predominantly on acute medicine. Further, within acute medicine, some SDEC services focus on specific pathways, for patients presenting with particular conditions, such as low risk chest pain, cellulitis, or low risk pulmonary embolism, and exclude patients who do not fit such defined pathways.^4^ For selecting patients some services use a risk stratification tool such as the AMB (ambulatory care) Score, a simple seven element scoring tool, 5, 6, 7 the GAPS (Glasgow Admission Prediction Score), although questions have been raised as to the validity of these tools, ^8^ or the National Early Warning Score, based on physiological observations.^9^ Others take a more inclusive approach, taking all medical referrals, as long as clinically stable.^10^^,^^11^

This scoping review will focus on acute medical SDEC, as medical patients represent a significant proportion of emergency admissions in the NHS.^12^ Typically, up to 30% of these patients are discharged within 24 h of arrival, suggesting that admission avoidance, using SDEC services, would meet that need and reduce pressure on inpatient beds.^12^ Where necessary, SDEC patients can attend again on subsequent days, for further investigation or review, rather than being admitted. Some services are developing this observational further, using Virtual Wards and Hospital at Home,^4^^,^^13^ but these services are beyond the scope of this review, being beyond the immediacy of same day care.

An emerging evidence base suggests that ambulatory services in the UK may improve patient experience, reduce hospital admissions, infection risk and deconditioning and offer cost savings.^14^ However, ambulatory care provision has been heterogeneous, whereas SDEC now has a national strategy, with core requirements,^3^ although local variations remain significant, in terms of speciality coverage; and some AEC services have simply been rebranded as SDEC. Embedding the SDEC national strategy should guide future service development in a more cohesive manner, leading to a more homogenous service across NHS regions. This in turn will support future research, as comparisons between services will be less varied.

Given that the SDEC model has been operational for a relatively short period of time, a review of the evidence base is indicated to establish what research supports the model. It is not uncommon for new policies to be rolled out, based on small pilot studies, in a top-down approach from the Department of Health and Social Care, as political agendas often drive cost-savings and efficiency, given the ever-increasing demand on the NHS. 15, 16, 17 It remains to be seen whether medium- and long-term costs savings are produced by SDEC services, given the requirement for additional pathways, estate, and staffing, or whether this approach simply delays admission and/or increases severity of illness at presentation, particularly in the older, frail population.

Establishing existing evidence underpinning the SDEC model will identify gaps that require investigation through further research. Initial searches suggest that there is little published literature to date, so a scoping review was chosen as the appropriate type of literature review to map emerging evidence. 18, 19, 20, 21 The Joanna Briggs Institute (JBI) methodology for scoping reviews^22^ was chosen to provide structure, and to support the development of a comprehensive overview of available evidence in relation to adult medical SDEC.

A preliminary search of MEDLINE, the Cochrane Database of Systematic Reviews, JBI Evidence Synthesis, and PROSPERO was undertaken to ascertain if this topic had been investigated previously, and no current, or in progress, systematic reviews or scoping reviews on the topic were identified.

## Review question

What is the existing evidence base for adult medical Same Day Emergency Care in UK hospitals?

### Objective

To determine the existing evidence base in relation to UK-based medical SDEC.

### Types of sources

This scoping review considers all study designs, texts, and opinion papers for inclusion.^22^

## Methods

The methods follow the JBI recommended structure of: Search strategy; Study/Source of Evidence selection; Data Extraction; Data Analysis and Presentation,^22^ followed by a narrative summary using a thematic approach.

A variety of evidence synthesis methodologies are available, including a range of different types of review, including systematic reviews, mixed methods reviews, realist syntheses, all aiming to inform policy, practice and/or further research, through rigorous, explicit, and systematised methods.^23^ If answers to clinically meaningful questions or to produce practice guidance are required a systematic review is likely to be the preferred review option, whereas to identify the types of evidence available in a particular area, to identify key characteristics or factors, and to identify and analyse knowledge gaps, a scoping review is likely to be more suitable.^20^ A scoping review offers an initial evaluation of the possible volume and range of existing literature; its primary goal is to recognise the nature and breadth of evidence for policy or practice, in contrast to systematic reviews.^21^

Considering the objectives of this review, a scoping review was selected as the appropriate methodological approach to systematically identify and map the evidence, across a wide range of sources, relating to SDEC. This assists in clarifying the evidence base and identifies key characteristics and/or factors relating to SDEC, as well as noting and analysing gaps in knowledge to develop future research. Critical appraisal of individual sources of evidence is not normally required for scoping reviews, hence no subsequent critique is offered.^22^

The guidance provided by the JBI Manual for Evidence Synthesis, Chapter 11: Scoping Reviews^22^ offers a robust, structured framework, that builds on and refines previous iterations of scoping review frameworks.^21^ This guidance recommends that an a priori protocol is required to support a systematic scoping review, accordingly a protocol was developed, registered,^24^ and published.^25^ In further alignment with the JBI guidance the Preferred Reporting Items for Systematic Reviews and Meta-analyses extension for scoping review (PRISMA-ScR) guideline has been utilised for this review.^21^^,^^23^^,^^26^

### Search strategy

The search aimed to locate both published and unpublished studies. An initial limited search of MEDLINE and CINAHL, via EBSCOhost, was undertaken to identify articles on the topic. The words contained in titles and abstracts of relevant articles were used to develop a full search strategy (Table 1), adapted for each database and/or information source, including all keywords. The reference lists of all included sources of evidence were screened for additional studies.^22^

**Table 1:** Search strategy.

Keywords: Same AND Day AND Emergency AND Care OR SDEC OR Ambulatory AND Emergency AND Care OR AEC, using Boolean operators
Eligibility Criteria:
*Participants*
Adults (over 18)
*Concept*
Same Day Emergency Care model of care delivery and acute medicine specialty, excluded condition specific papers and papers relating to other specialties
*Context*
NHS hospitals in the UK

Studies published in English were included, as this review focuses on UK-based SDEC services. Studies published in the past five years were included, due to the recent initiation of the SDEC model, the specific focus on SDEC, the general timeline for operation of SDEC, and the wide-ranging differences between the former heterogenous AEC services. Papers were only included where full text versions were immediately available. (Table 2: Inclusion and Exclusion Criteria).

**Table 2:** Inclusion and exclusion criteria.

*Inclusion criteria:*	English language, within the last 5 years, full text available, UK only, Adults (over 18), acute medicine specialty.
*Exclusion criteria*:	Foreign language, older than 5 years, no full text available, non-UK papers, paediatrics (under 18), specialties other than acute medicine.

The databases searched include EMBASE, MEDLINE and CINAHL, via EBSCOhost, during August 2023. Sources of unpublished studies, policies and grey literature searched comprised Google Scholar, the Cochrane Library, TRIP database, ProQuest Dissertations and Theses Open, and the Health Management Information Consortium.

### Study/source of evidence selection

**Fig. 1:**
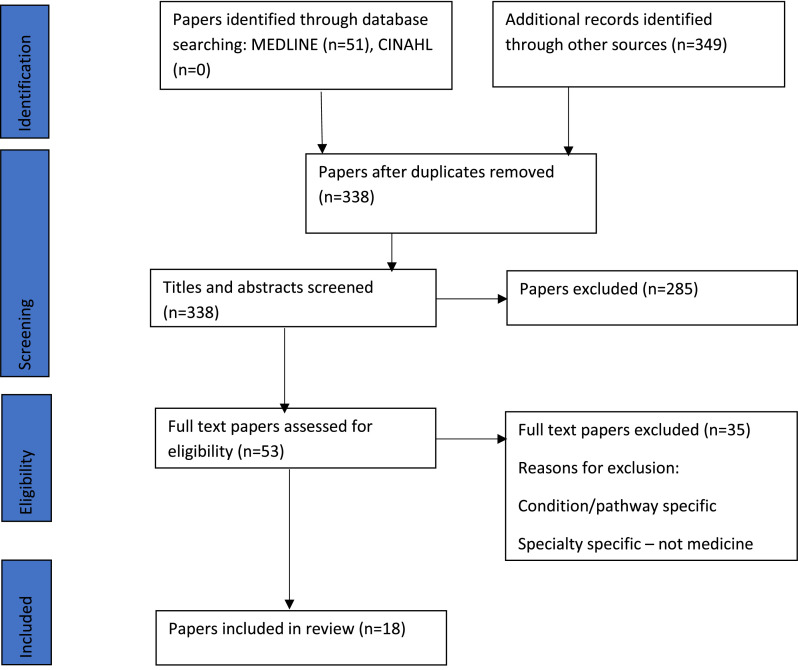
Flow diagram of search.

Following the search, all identified citations were collated and uploaded into EndNote Web^27^ and duplicates removed. Titles and abstracts were screened by one reviewer against the inclusion criteria. Potentially relevant papers were retrieved in full. The full text of selected papers was assessed, against the inclusion criteria, by two reviewers. Reasons for exclusion of papers, at the full text stage, that did not meet the inclusion criteria were recorded and are reported.^22^ The results of the search and the study inclusion process are reported and presented in a PRISMA flow diagram (Fig. 1).

### Data extraction

Data was extracted from included papers by two reviewers, using a data extraction tool based on the JBI data tool; differences of opinion were discussed until consensus was reached.^22^ Extracted data includes specific details about the participants, concept, context, study methods, and key findings relevant to the review question.

### Data analysis and presentation

**Fig. 2:**
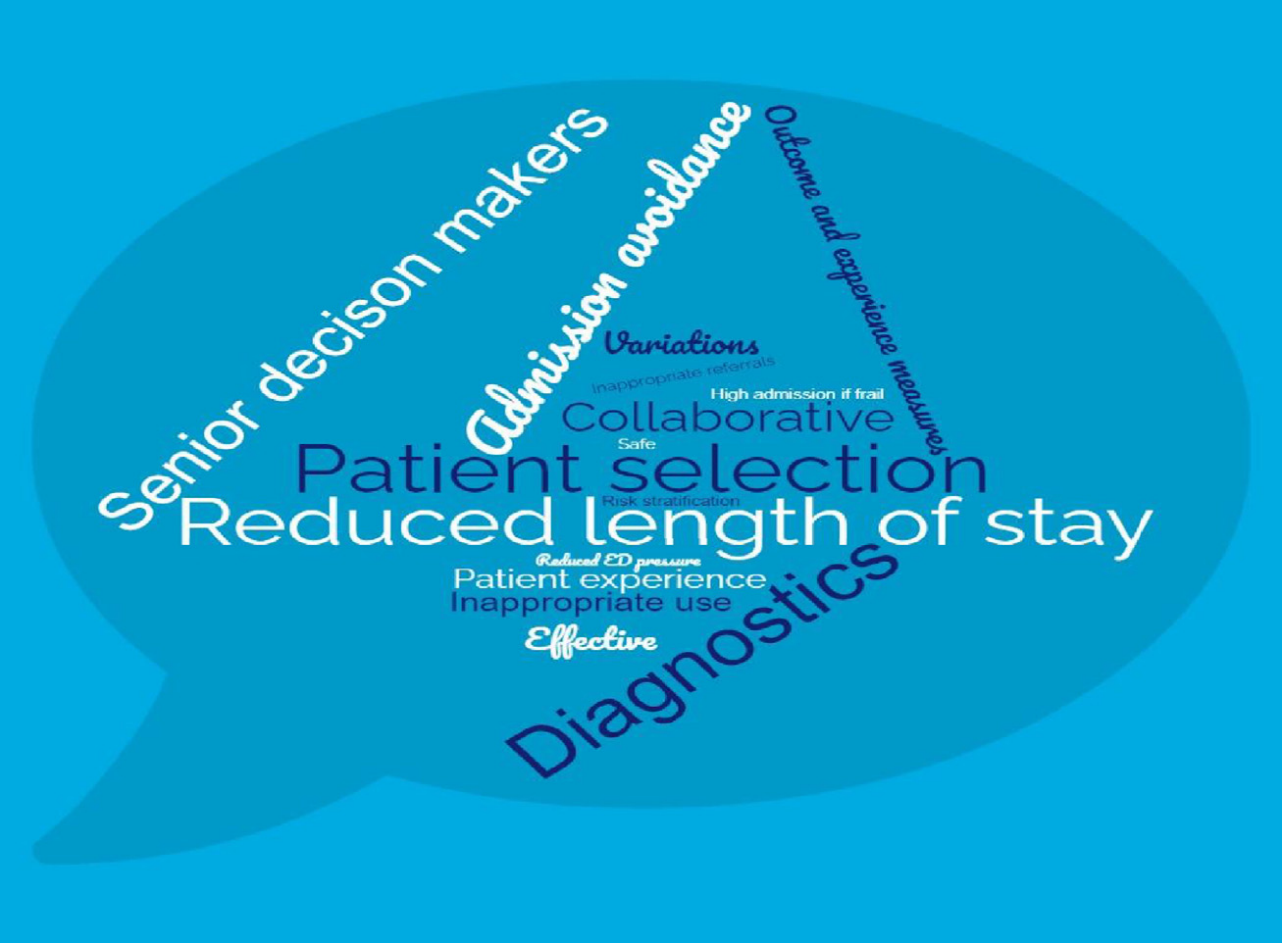
Weighted key concepts.

Data from the extraction tools was summarised and is presented in tabular form in Table 3. Types of papers and key concepts were identified and are presented in Table 4. A word cloud was generated via a weighted list of key theme frequencies to illustrate key concepts (Fig. 2).^28^

**Table 3:** Table of papers.

Reference	Type of Study/Paper	Study Period/Size (if applicable)	Outcomes Presented (if applicable)	Conclusions Drawn or Key Themes
^10^	Report on activity and outcomes of Same Day Emergency Care in a large medical ambulatory care by default model (observational cohort study in a single centre)	38 months 20,125 patient spells (16,497 patients)	42% of medical patients seen on ambulatory pathway in last 6 months of study 74.8% completed care in a single visit, 14% visits on two consecutive days Readmission within 30-day rate 7% (nationally 14.4% for inpatients) Mean age of SDEC patients 59.5 vs 67.3 on non-ambulatory pathway Diagnoses Conversion to admission rate 6.9% vs 18% on non-ambulatory pathway 30-day mortality 1.6% vs 6.9% on non-ambulatory pathway Also offer hospital at home and virtual ward facilities	Ambulatory approach is safe and effective Increasingly patients were managed on an SDEC pathway rather than by admission, so reduced inpatient bed requirements Outcomes demonstrate the value and safety of an inclusive, clinician-driven approach to patient selection
^38^	Audit report on pre- and post-implementation of an ambulatory emergency clinic, within an acute medical unit, based on SDEC principles Objective to determine any factors leading to delays in assessment, investigation, management & discharge Used PDSA service improvement methodology to address identified factors: Moving a medical registrar to SDEC in the evening Single point of triage GP patients sent to ED moved to SDEC Access to trolleys, not just chairs	Pre SDEC, October 2018, prospectively from January 2019, but no details as to how long data was collected post-implementation of SDEC, no information on sample size, single centre	Reduction in time to senior medical review from 3 h 19 minutes to 2 h 56 min Reduction in decision to discharge time from 3 h 28 minutes to 2 h 30 min Unified triage (bloods/investigations requested by nurses at triage so all information ready when patient seen by doctor), improved time to triage from 39 min to 20 min. Pre-SDEC 50% of GP patients seen by medics in ED & 9% seen in ambulatory area, post-SDEC 47% seen there & only 7% seen in ED	Reduced patient journey time Reduced numbers in ED
^42^	Prospective observational study in an SDEC for older patients, single centre	533 patients, August to December 2015	Among 533 patients (median (IQR) age = 81 (68–87), 315 (59%) female), 453 (86%) were living at home but 283 (54%) required some form of care and 299 (56%) had Barthel<20. Falls, urinary incontinence and dementia affected 81/189 (43%), 50 (26%) and 40 (21%) of those aged >85 years.” Severe illness was present in 148 (28%) with broadly similar rates across age groups. Overall, 210 (39%) patients had a hospital admission within 30-days with higher rates in older patients: 96 (87%) of < 65 years remained on an ambulatory pathway versus only 91 (48%) of ≥ 85 years (p < 0.0001). Factors independently associated with hospital admission were severe illness (SIRS/point, OR = 1.46, 95% CI = 1.15–1.87, p = 0.002) and markers of frailty: delirium (OR = 11.28, 3.07–41.44, p < 0.0001), increased care needs (OR = 3.08, 1.55–6.12, p = 0.001), transport requirement (OR = 1.92, 1.13–3.27), and poor nutrition (OR = 1.13–3.79, p = 0.02).	Even with MDT approach, rates of hospital admission in those with severe illness and frailty were high
^40^	Service review – retrospective medical notes audit Pre and post SDEC (July 2013/July 2014)	Two acute sites, one trust 191 patients pre-SDEC 344 SDEC	SDEC patients had fewer diagnostic tests All SDEC patients discharged opposed to 2/3 discharged the same day pre-SDEC	Improved admission avoidance Reduced diagnostics (more targeted) Increased efficiency
^39^	Report on direct streaming from ED to SDEC, with four pathways	4-day period in September 2019	33 patients, only two admitted, so 93.93% discharged Average waiting time for ED 5 h 44 min, compared to 49 min in SDEC	Factor leading to unnecessary admissions was traditional referral process between ED and medicine
^43^	Audit of referrals to SDEC within an acute medical unit, 1 week period in 2018, prior to new medical unit opening, and re-audit afterwards in 2019 Education provided to medical team in between	Pre-SDEC 118 referrals SDEC 88 referrals	Pre-SDEC: 36% from acute medical team, 28% ED, 24% GPs, 12% inpatient wards for post-discharge review 31% of referrals rejected by consultant (majority from GPs, but 28% from medical on-call team) 30% referred onto other specialties/clinics Post-SDEC rejected referrals 18%, onward referral 19%, increase in post-discharge referrals to 32%, increase in referrals to follow up blood tests to 28%	Large number of inappropriate referrals Medical hot clinics would reduce inappropriate referrals Consultants should follow up own inpatient results, not refer to SDEC
^30^	Review article			Patient selection key Early senior decision making 30% of patients can be managed in SDEC Improved patient experience Need clear pathways and processes
^11^	Review article			Process driven, rather than condition specific pathways Ambulatory by default recommended Acute generalists, including advanced nurse practitioners, acute medicine, ED, or GP clinicians Rapid access to diagnostics key Risk inherent – identifying safe discharges Outcome & experience metrics are needed
^44^	Opinion piece	Refers to national audit (SAMBA)	Audit of 141 units – 96% had SDEC service, 45% of these used these as escalation beds at times of high demand,	SDEC services can be closed by bed pressures in times of high demand, compounding capacity issues
^45^	Opinion piece	Refers to SAMBA audit 2021	98% of 158 units (hospitals) had some form of SDEC of 158 hospitals, only 22% of patients received their medical assessment & treatment there, this was the same as 2019 and is below the 30% target set by NHS England	Inappropriate use of SDEC & lack of investment
^33^	Review article on acute medical SDEC			Variation in SDEC service design and patient selection methods within acute medicine
^4^	Article on the possibilities for acute medical care in the future			Describes key aspects of successful SDEC units and how these fit into acute medical services Highlights significant variations in provision
^1^	Policy			Definition of SDEC SDEC patient selection and streaming Proposed metrics
^2^	Policy			Reforms to hospital emergency care – SDEC Every hospital with a type 1 ED will have an SDEC model, to be embedded during 2019/2020 SDEC 7 days a week, 12 h a day minimum
^3^	Same Day Strategy			Seven key themes: Staffing must be safe and sustainable Access to SDEC available to all external stakeholders (111, ambulance, primary care etc.) Monitoring & evaluation of activity to be standardised Estate redesign Diagnostics & testing capacity for early decision making Alternative to admission where appropriate Compassionate leadership to change culture
^29^	Position statement			Endorsing the provision of SDEC services Benefits of effective SDEC: Reducing unwarranted variation in care pathways, streamlining the patient journey, improved patient & staff satisfaction, reducing admissions, improving flow
^32^	Policy			Toolkit for ED physicians Early and appropriate streaming needed Improves flow Collaborative working with acute specialties needed Rapid access to diagnostics key Performance measures needed to assess impact, quality & efficiency
^31^	Review article			Early access to senior decision makers needed Opening hours matching demand Access to diagnostics needed Close collaboration with clinical services needed Patient selection Risk stratification tools Highlights different models of SDEC – pathway vs process, push vs pull

**Table 4:** Results.

Study/article characteristics	n/18(%)			
Observational cohort study	2 (11.1)			
Audit	4 (22.2)			
Review articles	4 (22.2)			
Opinion pieces	2 (11.1)			
Future developments	1 (5.6)			
Policies	3 (16.7)			
Strategy	1 (5.6)			
Position statement	1 (5.6)			

Key concepts	n/3 (%)	n/9 (%)	n/6 (%)	n/18 (%)
	Policy	Reviews	Studies	Overall

Safety			1 (16.7)	1 (5.6)
Effectiveness			2 (33.3)	2 (11.1)
Admission avoidance	2 (66.7)		3 (50)	5 (27.8)
Reduced length of stay	3 (100)		2 (33.3)	5 (27.8)
Reduced pressure on ED			1 (16.7)	1 (5.6)
High admission if frailty			1 (16.7)	1 (5.6)
Inappropriate referrals			1 (16.7)	1 (5.6)
Senior decision makers	1 (33.3)	3 (33.3)		4 (22.2)
Access to diagnostics	2 (66.7)	2 (22.2)	1 (16.7)	5 (27.8)
Collaborative working	2 (66.7)	1 (11.1)		3 (16.7)
Patient selection	3 (100)	6 (66.7)	1 (16.7)	10 (55.6)
Risk stratification		1 (11.1)		1 (5.6)
Outcome/experience measures needed	3 (100)	1 (11.1)		4 (22.2)
Patient experience	1 (33.3)	1 (11.1)		2 (11.1)
Inappropriate use		2 (22.2)		2 (11.1)
Variations	1 (33.3)	1 (11.1)		2 (11.1)

## Discussion

NHS England and NHS Improvement^2^ mandated the implementation of SDEC in every hospital with an ED during 2019/2020 and is endorsed by The Royal College of Emergency Medicine (RCEM) and the Society for Acute Medicine,^29^ this position statement espouses the benefits of SDEC as reducing unwarranted variation in care pathways, streamlining the patient journey, improved patient, and staff satisfaction, reducing admissions, and improving flow. However, as indicated in the papers identified (Table 4), there is limited research relating to SDEC as a model of care, but several themes became apparent during analysis.

### Factors required for success

Identifying factors required for success in operating SDEC is considered by several review articles and policies.

#### Patient selection—pathways vs process driven

Selecting the right patients for SDEC is key with 30% of patients presenting with an acute medical issue being suitable for management on SDEC.^30^ The need for clear pathways and processes for ensuring that the right patients are referred to SDEC is evident.^1^^,^^30^^,^^31^ Cottrell *et al.*^11^ recommend a process driven, ambulatory by default mind set, rather than condition specific pathways, a view supported by RCEM.^32^ The variation in SDEC service design and patient selection methods is highlighted by Atkin *et al.*^33^ and Dean *et al.*^4^; although the Same Day Strategy^3^ should lead towards standardisation of services in the future. Indeed, this strategy promotes widening access, stating that SDEC should be available to all stakeholders, including primary care, ambulance services, out-of-hours services, and not just be considered for reassigning ED patients.

#### Early senior decision making and collaborative working

Having a senior decision maker in SDEC facilitates prompt review and allows early management and discharge decisions to be made.^11^^,^^30^^,^^31^ Although Cottrell *et al.*^11^ go further and acknowledge that acute generalists, including advanced practitioners, acute medicine and ED doctors or GPs can offer expanded options for staffing SDEC with senior clinicians. This is notably the only mention evident of the contribution of advanced practitioners within SDEC in the literature. This is surprising, given workforce challenges and that the need for, and value of, advanced practitioners has been recognised for some years,^34^ whilst the need for safe and sustainable staffing,^3^ and collaborative working across specialties and traditional boundaries is recognised by some.^31^^,^^32^

#### Rapid access to diagnostics

To facilitate assessment of patients within SDEC, access to rapid diagnostics, in the same timeframe as the ED, is required, this is a key factor for success, as without diagnostics decision-making for safe discharge is problematic.^11^^,^^31^^,^^32^ Achieving rapid access to diagnostics requires negotiation and potentially investment in diagnostic services to increase capacity. However, the growing ability of clinicians to undertake diagnostic medical ultrasound^35^ will relieve some of the pressure on the diagnostic team if equipment is available on SDEC. Such point of care testing in SDEC also offers opportunities for rapid results on other investigations such as troponin and D-dimer,^33^ but again requires investment.

### Measures of success

There is a lack of clarity as to what metrics should be collected on SDEC to determine what success looks like. NHS Improvement and the Ambulatory Emergency Care Network^1^ proposed metrics but these have not been widely adopted. RCEM^32^ highlighted the importance of developing performance measures to assess impact, quality, and efficiency. The Same Day Strategy^3^ commits to standardisation of monitoring and evaluation of process activity. NHS England^36^ are committed to rolling out the ‘ECDS’ (Emergency Care Data Set) to record SDEC activity, separately from admitted patient care data, but contracting issues are posing a barrier and no implementation date has been set. Data is being collected on SDEC, but these are process data rather than clinical outcome data, for example: number of attendances, time spent on SDEC, admission and discharge within six hours, percentage of virtual consultations, and average weekly opening hours.^37^

In a single centre retrospective study,^10^ hospital data on 16,497 patients was analysed for an adult medical SDEC service, seeking to establish activity and outcomes. In the last calendar year studied, the conversion rate from SDEC to inpatient admission was 12%, the 30-day readmission rate was 6.9% (18% for the admitted pathway). Across the 3-year period, 30-day mortality was 1.6%. There was a reduction in inpatient bed requirements. The conclusion drawn was that the SDEC approach is safe and effective, and within the limitations of a single-centre study, thus supports SDEC services. Using conversion to inpatient admission, 30-day readmission and 30-day mortality seem reasonable safety metrics, and the latter two permit comparison with inpatient admission data.

A comparative single centre study of an SDEC service within an acute admissions unit evaluated the period before and after the SDEC service opened to identify its impact.^38^ Reducing pressure on the ED and medical take, reducing time to clinical review and discharge were found to be benefits of improving the patient pathway, which are reasonable metrics on effectiveness. Reducing ED pressure was also a benefit highlighted^39^ in a 4-day study looking at 33 patients taken directly from ED, after initial assessment, with two admissions resulting.

Baker^40^ considered discharge and admission avoidance, and demonstrated improved admission avoidance, more targeted use of diagnostics and improved efficiency in a service review pre and post SDEC implementation. A short audit on direct streaming from ED to SDEC, demonstrated shorter length of stay and a high discharge rate for those seen within SDEC, it was identified that the traditional referral process between ED and acute medicine led to unnecessary admissions and delays in the patient journey, that can be ameliorated by rapid streaming to SDEC.^39^

It seems that clinically relevant outcomes are largely unknown when SDEC services are considered. As discussed by Atkin *et al.,*^3^ evidence of safety and positive impact on patient care is limited. Their literature review found some evidence, but this was generally confined to a few specific conditions, such as pulmonary embolus and low risk community acquired pneumonia. They conclude that no robust studies exist to demonstrate benefit for most conditions, and no studies of cost effectiveness have been undertaken in relation to SDEC. This view is shared by Cottrell *et al.*^11^ who advise that outcome and experience metrics are needed.

Thompson and Connolly^30^ discuss how patient experience is improved by managing care on SDEC, rather than through inpatient admission. But notably there are no published studies on the patient experience of SDEC. Patient satisfaction is a key measure of quality, the NHS England Experience of Care Group has recently undertaken a co-production project to develop Quality Markers and Metrics for SDEC,^41^ a project that the first author of this review was involved with; this collaborative project should lead to benchmarking across SDEC services and generate service improvement initiatives.

### Barriers to success

Elias *et al.*^42^ undertook a single centre prospective observational cohort study, in a multidisciplinary SDEC, with 533 older frail patients, aiming to understand factors associated with admission to bed-based care, concluding that conversion to admission is high in this cohort; this suggests that SDEC may not be effective in this population and illustrates the importance of selecting the correct cohort of patients for SDEC.

An audit of referrals pre and post the implementation of an SDEC, within an acute medical unit, demonstrated many inappropriate referrals, some requiring an alternate specialty review other than acute medicine, an expectation that SDEC would follow up inpatient tests post-discharge and a lack of medical ‘hot clinics’ i.e. capacity to see discharged patients within a few days.^43^ Standardisation and education on the purpose of SDEC will ameliorate lack of understanding amongst professionals, and robust standard operating procedures will support clinicians in declining inappropriate referrals. Organisations should consider whether acute medicine hot clinics should be implemented to follow up post-discharge patients, or whether these patients should be seen in SDEC, given that the hospital contract requires trusts to review these patients rather than discharge them for general practice review within days.

The risk of SDEC services being derailed by trusts repurposing these areas as inpatient areas during times of high demand remains a threat to SDEC provision.^44^ This concern is supported by Mahase,^45^ citing data from the national Society of Acute Medicine Baseline Audit that only 22% of patients received their medical assessment and treatment in an SDEC setting. The underlying reasons were lack of investment in SDEC services and inappropriate use of SDEC, including using the area for escalation and converting it to a bedded area for inpatients in times of high demand.

The Same Day Strategy^3^ requires suitable estate to facilitate the implementation of SDEC services within trusts. However, the availability of space within existing estate for the development of SDEC may be a barrier, along with the requirement for investment, as there are competing pressures on resources, and capital investment can be difficult to secure.

### Areas where no papers were identified

There were no studies exploring patient experience of SDEC, nor of the workforce supporting SDEC services, such as the use of advanced practitioners in addition to, or instead of, medical staff.

## Limitations

Scoping reviews present and summarise the papers identified; critical appraisal and risk of bias assessments are not required in scoping reviews; some methodologists suggest that this is a limitation, as they cannot be used as evidence to guide practice but are instead designed to identify gaps in the literature. In hindsight, limiting the search to the previous five years may have been too short, and resulted in valuable learning from the former iteration of SDEC – AEC, being excluded, but earlier papers have been used in this paper's narrative for contextualisation.

## Recommendations and conclusions

Based on the features of SDEC services emerging from this review, it is recommended future SDEC research could usefully consider:

• Clinical outcomes of medical patients attending SDEC, compared to those admitted to hospital for a short stay. Specific measures could include conversion to admission rate, readmission within 30-days, 30-day mortality.
• Patient experiences of SDEC.
• Workforce studies – is there a variance in clinical outcomes or patient experience in services led by advanced clinical practitioners vs. senior medical staff, and associated cost-effectiveness of different staffing models.

SDEC is now a mandated requirement within the NHS in the UK and requires an underpinning evidence-base. The scarcity of literature in relation to the quality, safety, and effectiveness of SDEC highlights the need for further study. Whilst different organisations operate SDEC in variable ways, as to be expected in the complex systems within the NHS, more standardisation and reduction in variation will increase the ability to evaluate specific SDEC services and make comparisons between SDEC services. Therefore, there is a pressing need for SDEC specific research within the UK, such as this scoping review has noted.

## Contributions

SD proposed the initial idea for reviewing the topic of SDEC. JB proposed the idea for utilising a scoping review approach. SD wrote the initial drafts of the paper and undertook the initial preliminary review of the literature for this paper. JB commented on and revised drafts of this paper and formatted the pre-submission draft.

## Declaration of competing interest

There are no conflicts of interest in this project.
